# SMRT sequencing of full-length transcriptome of flea beetle *Agasicles hygrophila* (Selman and Vogt)

**DOI:** 10.1038/s41598-018-20181-y

**Published:** 2018-02-02

**Authors:** Dong Jia, Yuanxin Wang, Yanhong Liu, Jun Hu, Yanqiong Guo, Lingling Gao, Ruiyan Ma

**Affiliations:** 1 College of Agriculture, Shanxi Agricultural University, Taigu, 030801 China; 2 College of Life Science, Shanxi Agricultural University, Taigu, 030801 China; 3 CSIRO Agriculture and Food, Centre for Environment and Life Sciences, Wembley, Western Australia 6014 Australia

## Abstract

This study was aimed at generating the full-length transcriptome of flea beetle *Agasicles hygrophila* (Selman and Vogt) using single-molecule real-time (SMRT) sequencing. Four developmental stages of *A. hygrophila*, including eggs, larvae, pupae, and adults were harvested for isolating total RNA. The mixed samples were used for SMRT sequencing to generate the full-length transcriptome. Based on the obtained transcriptome data, alternative splicing event, simple sequence repeat (SSR) analysis, coding sequence prediction, transcript functional annotation, and lncRNA prediction were performed. Total 9.45 Gb of clean reads were generated, including 335,045 reads of insert (ROI) and 158,085 full-length non-chimeric (FLNC) reads. Transcript clustering analysis of FLNC reads identified 40,004 consensus isoforms, including 31,015 high-quality ones. After removing redundant reads, 28,982 transcripts were obtained. Total 145 alternative splicing events were predicted. Additionally, 12,753 SSRs and 16,205 coding sequences were identified based on SSR analysis. Furthermore, 24,031 transcripts were annotated in eight functional databases, and 4,198 lncRNAs were predicted. This is the first study to perform SMRT sequencing of the full-length transcriptome of *A. hygrophila*. The obtained transcriptome may facilitate further exploration of the genetic data of *A. hygrophila* and uncover the interactions between this insect and the ecosystem.

## Introduction

Alligator weed *Alternanthera philoxeroides* (Mart.) (Amaranthaceae) that originated from South America^1,2^, was introduced into China in the 1930 s. In China, *A. philoxeroides* is an important invasive species and has resulted in ecological and economic damage^3^. To control *A. philoxeroides* infestations, the flea beetle *Agasicles hygrophila* (Selman and Vogt) (Coleoptera: Chrysomelidae) was introduced as a biological control agent^4^. The use of *A. hygrophila* as a biological control of *A. philoxeroides* is acknowledged to be the world’s first successful example of aquatic weed control^5^.

Study has reported that the physiological adaptations of biological control agents determine where and when they will be successful^6,7^. Host specificity and potential host shifting are considered as the most important factors for the evaluation of *A. hygrophila* in many countries where it is introduced for biological control of *A. philoxeroides*^8^. Due to the good performances in host selection and ecological adaptation, *A. hygrophila* has become a desirable model insect for the investigation of the relationships between insects and plants, as well as insects and ecosystem. Currently, the genome and transcriptome information of *A. hygrophila* has not been investigated, which hinders the study of the molecular mechanisms underlying the interaction between *A. hygrophila* and host plant and ecosystem.

Transcriptome could reflect the type and number of intracellular genes and reveal the physiological and biochemical processes at a molecular level^9^. Several technologies have been applied for transcriptome sequencing. Among these, short-read transcriptome sequencing has become a powerful tool for the description of gene expression levels^10,11^. However, most of these technologies are incapable of assembling full-length transcripts because of the shortness of sequencing reads, which necessitates efforts for exploring other technologies. Thus, single-molecule real-time (SMRT) sequencing (Pacific Biosciences of California, Inc., CA, USA) is developed^12^, which overcomes the limitation of short-read sequences by enabling the generation of kilobase-sized sequencing reads^13^. The full-length transcriptome can be used to analyze the alternative splicing events, and the primary-percusor-mature RNAs structures, which help better understanding the RNA processing.

In this study, SMRT sequencing was performed to generate full-length transcriptome of *A. hygrophila*. Based on the obtained transcriptome data, we performed alternative splicing analyisis, simple sequence repeat (SSR) analysis, coding sequence prediction, transcript functional annotation, and lncRNA prediction. This study may be a valuable resource for further investigation of *A. hygrophila*.

## Materials and Methods

### Insects and host plants

The flea beetles, *A. hygrophila*, were obtained from South China Agricultural University (Guangdong, China) and were maintained in the insectary of Shanxi Agricultural University (Shanxi, China) under controlled conditions of 25 ± 1°C, with a light: dark photoperiod of 14:10 h and 80 ± 5% relative humidity. The insects were reared for several generations to obtain an experimental population with a consistent genetic background.

The host plant *A. philoxeroides* was collected from a field greenhouse at Yuhuan County, Zhejiang, China and was planted in the greenhouse of the biosafety and biological control research base at Shanxi Agricultural University.

### Sample processing

The eggs laid by *A. hygrophila* within 24 h, first- to third-instar larvae starved for 24 h, pupae (2–6 days old), and newly emerged adults starved for 24 h were gathered and rinsed three times in precooled normal saline. Finally, 0.3 g of eggs, 0.8 g of larvae, 3.8 g of pupae, and 6.2 g of adults were harvested and frozen in liquid nitrogen for further experiments.

### RNA sample preparation

Total RNA samples (at four different developmental stages) were isolated using the RNeasy Plus Mini Kit (Qiagen, Valencia, CA, USA). RNA degradation and contamination were monitored using 1% agarose gels. The purity and concentration of RNA were measured using the NanoDrop ND-1000 spectrophotometer (NanoDrop Technologies, Rockland, DE, USA) with a OD_260_/_280_ reading. The RNA integrity was assessed using the RNA Nano 6000 Assay Kit of the Agilent Bioanalyzer 2100 system (Agilent Technologies, CA, USA). The total RNA samples from four developmental stages were mixed together for the following experiments.

### Library preparation and SMRT sequencing

mRNA was purified from 3 µg of mixed total RNA using poly (T) oligo-attached magnetic beads. Fragmentation was conducted using divalent cations under elevated temperatures in the NEBNext First Strand Synthesis Reaction Buffer (5×). The SMART PCR cDNA Synthesis Kit (Clontech, CA, USA) was used for synthesizing full-length cDNA. Remaining overhangs were converted into blunt ends via exonuclease/polymerase activities. After adenylation of the 3′ ends of the DNA fragments, NEBNext Adaptor with a hairpin loop structure was ligated to prepare for hybridization. BluePippin® (Sage Science, Beverly, MA, USA) was used for size selection of the full-length cDNA and for building libraries of differently sized cDNA. The generated cDNA was then re-amplified using PCR, and the fragment size distribution was quantified using the Qubit fluorometer (Life Technologies, Carlsbad, CA, USA). The quality of the libraries was assessed using the Agilent Bioanalyzer 2100 system. SMRT sequencing was performed using the Pacific Biosciences’ real-time sequencer using C2 sequencing reagents.

### Next-generation sequencing

Total RNA (5 μg) was digested by using DNase I (NEB, Frankfurt, Germany). The sample was purified with Agencourt RNAClean XP Beads and fragmented into 130–170 nt. First-strand cDNA was generated by First Strand Master Mix and Super Script II reverse transcription (Invitrogen). Then second-strand cDNA was synthesized using Second Strand Master Mix. After end repairing, adding A and adaptor ligation, several rounds of PCR amplification with PCR Primer Cocktail and PCR Master Mix were performed to enrich the cDNA fragments. The final library is quantitated by using the Agilent 2100 bioanalyzer instrument, and real-time quantitative PCR. The qualified libraries was sequenced pair end on the Illumina HiSeq. 2000 System.

### Preprocessing of SMRT reads

Raw SMRT sequencing reads were processed by removing polymerase reads that were <50 bp and had quality of <0.75, obtaining the clean reads. The obtained clean reads were processed into error-corrected reads of inserts (ROIs) with parameters of full passes of ≥0 and quality of >0.75. The ROI reads with both the 5′ and 3′ primer sequences and a poly(A) tail present were considered to be full-length transcripts. During the processes of library preparation, the chimeric sequences formed by the direct linkages of two cDNA template strands due to the low concentrations of adapter or SMRTbell are called artificial chimeric sequences. The non-chimeric sequences in the full-length sequence are called full-length non-chimeric (FLNC) sequences. The FLNC transcripts were determined by searching for the poly(A) tail signal and the 5′tail si cDNA primers in ROIs.

Iterative clustering for error correction (ICE) in SMRT analysis (v2.3.0)^12^ was used to obtain consensus isoforms by approaching clustering, and the full-length consensus sequences from ICE were refined using Quiver. Full-length transcripts with post-correction accuracy of >99% (high-quality isoforms) were generated for further analysis. Any redundancy in high-quality full-length transcripts was removed by CD-HIT^14^.

### Alternative splicing detection

RNA alternative splicing, occurring after a pre-mRNA transcript, is formed from template DNA, which results in a single gene coding for multiple proteins. During this process, particular exons of a gene may be included within or excluded from the final, processed mRNA produced from that gene^15^, which results in the proteins translated from alternatively spliced mRNAs containing differences in their amino acid sequence and biological functions. In this study, based on the obtained redundancy removed transcripts, we predicted the alternative splicing events. Briefly, all sequences were aligned to each other with BLAST^16^. The alignment results according with the following conditions were considered as alternative splicing events^17^:

- Both sequences lengths were more than 1000 bp, besides there should be two High-scoring Segment Pairs in the alignment;
- The alternative splicing Gap was greater than 100 bp, with at least 100 bp distance to the 3′/5′ end;
- All alternatively spliced transcripts allowed 5 bp overlap.

### Simple sequence repeat (SSR) detection

SSR, also known as microsatellite, is a tract of repetitive DNA in which certain DNA motifs (ranging in length from 2–13 base pairs) are repeated, typically 5–50 times^18^. Transcripts that were >500 bp were selected for SSR analysis using the MIcroSAtellite identification tool (MISA; http://pgrc.ipk-gatersleben.de/misa/http://pgrc.ipk-gatersleben.de/misa/)^19^. MISA can identify seven SSR types, namely mononucleotide, dinucleotide, trinucleotide, tetranucleotide, pentanucleotide, hexanucleotide, and compound SSR, by analyzing transcript sequences.

### Prediction of coding sequences

The coding sequences and corresponding amino acid sequences within the transcript sequences were predicted using TransDecoder (https://github.com/TransDecoder/TransDecoder/releases). TransDecoder could identify candidate protein-coding regions based on nucleotide composition, open reading frame (ORF) length, log-likelihood score, and (optional) Pfam domain content^20^.

### Functional annotation of transcripts

The obtained non-redundant transcript sequences were mapped to eight databases to obtain the annotation information of the transcript. These databases included NR^21^, Swiss-Prot^22^, Gene Ontology (GO; http://www.geneontology.org)^23^, Clusters of Orthologous Groups of proteins (COG; http://www.ncbi.nlm.nih.gov/COG)^24^, euKaryotic Ortholog Groups (KOG)^25^, Pfam (http://pfam.janelia.org/)^26^, evolutionary genealogy of genes: Non-supervised Orthologous Groups (eggNOG; http://eggnog.embl.de), and Kyoto Encyclopedia of Genes and Genomes (KEGG, http://www.genome.ad.jp/kegg/)^27^.

### lncRNA prediction

The most widely used methods for analyzing coding potential are Coding Potential Calculator (CPC)^28^, Coding-Non-Coding Index (CNCI)^29^, Coding Potential Assessment Tool (CPAT)^21^, and pfam protein structure domain analysis. In this study, lncRNAs were predicted by screening the coding potential of transcripts using these four methods above.

## Results

### SMRT sequencing data output

Bases on the Pacific Biosciences’ SMRT sequencing technology, 456,994 polymerase reads were generated. After preprocessing, 9.45 Gb of clean reads were obtained (Table 1). On the basis of the conditions of full passes of ≥0 and quality of >0.75, 335,045 ROIs were obtained (Table 2). In addition, 158,085 FLNC sequences were identified (Table 3).

**Table 1:** Polymerase reads sequence statistics.

Sample name	cDNA size	SMRT cells	Polymerase reads	Post-filter polymerase reads	Post-filter total number of subread bases	Post-filter number of subread	Post-filter subreads N50	Post-filter mean subread length
T01	0.5–1 K	2	300,584	158,893	3,021,283,493	3,163,026	938	955
T01	1–2 K	2	300,584	199,791	4,169,421,605	2,534,817	1,648	1,644
T01	2–6 K	1	150,292	98,310	2,263,026,061	801,048	2,835	2,825

cDNA size: insert fragment size of cDNA libraries; SMRT cells: the number of cells used for library construction; Polymerase reads: the number of polymerase reads sequences after sequencing; Post-filter polymerase reads: the number of polymerase reads sequences after filtration; Post-filter total number of subread bases: the number of subreads bases after filtration; Post-filter number of subread: the number of subreads after filtration; Post-filter subreads N50: subread N50 length after filtration; Post-filter mean subread length: average length of subread after filtration.

**Table 2:** Reads of insert (ROI) statistics.

Sample	cDNA size	Reads of insert	Read bases of insert	Mean read length of insert	Mean read quality of insert	Mean number of passes
T01	0.5–1 K	122,928	156,155,506	1,270	0.93	19
T01	1–2 K	142,751	279,590,957	1,958	0.92	12
T01	2–6 K	69,366	198,654,998	2,863	0.93	9

cDNA size: insert fragment size of cDNA libraries; Reads of insert: the number of ROI sequences; Read bases of insert: the total number of ROI bases; Mean read length of insert: average length of ROI; Mean read quality of insert: Quality value of ROI sequence; Mean number of passes: the mean sequencing depth of sequences in zero-mode wave.

**Table 3:** Full-length sequences statistics

Sample	cDNA size	Reads of insert	Number of five prime reads	Number of three prime reads	Number of poly-A reads	Number of filtered short reads	Number of non-full-length reads	Number of full-length reads	Number of full-length non-chimeric reads	Average full-length non-chimeric read length	Full-length Percentage (FL%)	Artificial concatemers (%)
T01	0.5–1 K	122,928	68,904	78,474	73,850	27,518	40,363	55,047	53,115	688	44.78%	3.51%
T01	1–2 K	142,751	76,467	85,726	83,554	23,630	55,571	63,550	63,051	1,225	44.52%	0.79%
T01	2–6 K	69,366	49,331	50,528	50,045	2,192	25,127	42,047	41,919	2,697	60.62%	0.30%

cDNA size: insert fragment size of cDNA libraries; reads of insert: the number of reads of insert (ROI) sequences; Number of five prime reads: the number of ROI sequences containing 5′ primer; Number of three prime reads: the number of ROI sequences containing 3′ primer; Number of poly-A reads: the number of ROI sequences containing poly-A; Number of filtered short reads: the number of filtered ROI of <300 bp; Number of non-full-length reads: the number of non-full-length ROI; Number of full-length non-chimeric reads: the number of full-length non-chimeric ROI; Average full-length non-chimeric read length: average length of full-length non-chimeric sequence; Full-length percentage (FL%): the percentage of full-length sequence in ROI sequence; Artificial concatemers (%): the percentage of full-length chimeric sequence in full-length sequence.

### Comparison of results of SMRT sequencing and next-generation sequencing

Most of the assembled contigs (70.41%) from next-generation sequencing were with length between 200–300 bp and only 2.31% were more than 2 kb. A total of 11,994 unigenes (25.99%) had length of 200–300 bp and 1,1981 unigenes (25.96%) had length of 300–500 bp. The comparison results between SMRT sequencing transcript and Illumina sequencing contig and unigene are shown in Table 4. Additionally, a total of 28,982 transcripts with total length of 48,811,662 bp were obtained from SMRT sequencing. For Illumina sequencing, 95,700 contigs and 46,151 unigenes (38,506,958 bp) were obtained (Table 5).

**Table 4:** Comparison results between SMRT sequencing transcript and Illumina sequencing contig and unigene.

Length distribution (bp)	SMRT sequencing transcript		Illumina sequencing assembled contig		Illumina sequencing assembled unigene	
	Number	Percentage	Number	Percentage	Number	Percentage
200–300	1	0.00%	67378	70.41%	11994	25.99%
300–500	1663	5.74%	11176	11.68%	11981	25.96%
500–1000	7331	25.30%	9331	9.75%	10993	23.82%
1000–2000	8796	30.35%	5600	5.85%	7472	16.19%
2000+	11191	38.61%	2215	2.31%	3711	8.04%

**Table 5:** Comparison of assembly indicators between SMRT sequencing transcript and Illumina sequencing contig and unigene

Indicator	SMRT sequencing transcript	Illumina sequencing assembled contig	Illumina sequencing assembled unigene
Total Number	28982	95700	46151
Total Length	48811662	35633777	38506958
N50 Length	2331	731	1312
Mean Length	1684.206128	372.348767	834.3688761

### Transcript clustering analysis

In total, 40,004 consensus isoforms were obtained, including 31,015 high-quality isoforms and 8,989 low-quality ones. The ICE clustering results are shown in Table 6. Finally, 28,982 transcripts were obtained after removing redundant sequences from the high-quality transcripts.

**Table 6:** Results of Iterative Clustering for Error Correction (ICE) clustering analysis.

Samples	Size	Number of consensus isoforms	Average consensus isoforms read length	Number of polished high-quality isoforms	Number of polished low-quality isoforms	Percent of polished high-quality isoforms(%)
T01	0–2 kb	22,147	1,036	18,973	3,174	85.67%
T01	2–3 kb	11,876	2,483	8,826	3,050	74.32%
T01	3–6 kb	5,548	3,647	3,212	2,336	57.89%
T01	>6 kb	433	8,785	4	429	0.92%

cDNA size: insert fragment size of cDNA libraries; Number of consensus isoforms: the number of consensus isoforms obtained from ICE clustering analysis; Average consensus isoforms length: sequence length of consensus isoform; Number of HQ isoforms: the number of high-quality transcripts; Number of LQ isoforms: the number of low-quality transcripts; Percent of HQ isoforms (%): percentage of high-quality transcripts in consensus isoform.

### Alternative splicing analysis

Total 146 alternative splicing events were identified, as shown in Supplementary Table S1. Additionally, since no reference genome is available for SMRT sequencing of transcriptome in *A. hygrophila*, we could not determine the types of alternative splicing events.

### SSR detection

A total of 27,318 sequences (48,121,807 bp) were subjected to SSR analysis, including 12,753 SSRs and 8,535 SSR-containing sequences. The number of sequences containing more than one SSR was 2,733, and the number of SSRs present in compound formation was 966. In addition, the numbers of mono nucleotides, di nucleotides, tri nucleotides, tetra nucleotides, penta nucleotides, and hexa nucleotides were 10,810, 922, 952, 59, 6, and 4, respectively.

### Prediction of coding sequences

**Figure 1:**
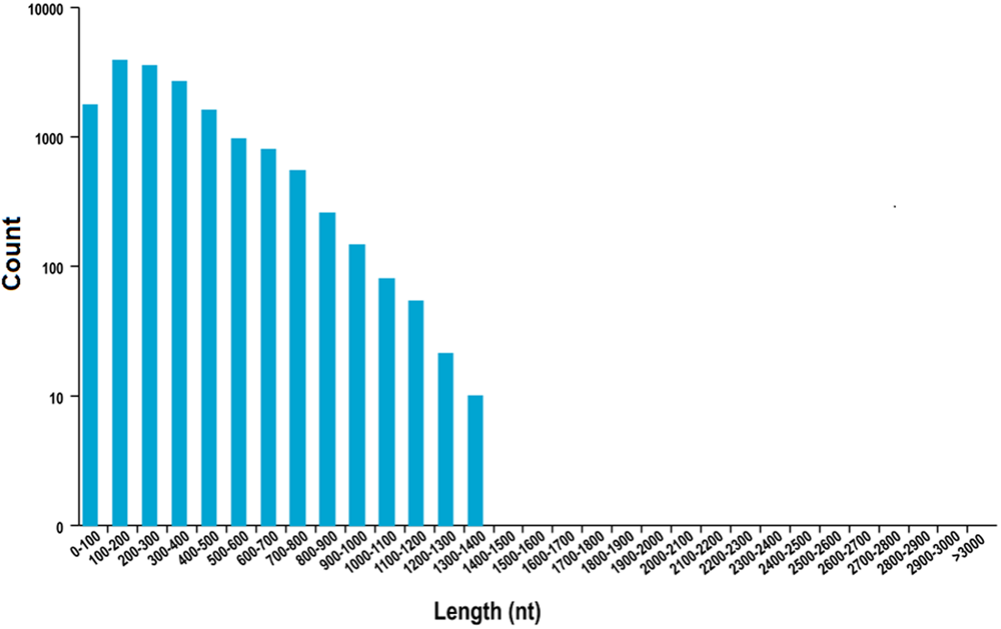
The distribution of the coding sequence lengths of the complete open reading frame. The x-axis represents the coding sequence length; the y-axis represents the number of predicted open reading frames.

Using TransDecoder, 24,040 ORFs were identified, which included 16,205 complete ORFs. The distribution of the coding sequence lengths of complete ORFs is shown in Fig. 1.

### Functional annotation of transcripts

In total, 8,292 transcripts were annotated in the COG database; 13,197 were annotated in GO; 12,592 in KEGG; 16,955 in KOG; 20,940 in Pfam; 15,025 in Swiss-Prot; 22,887 in eggNOG; and 23,793 in NR. Moreover, 24,031 transcripts were annotated in all of the eight databases.

### NR annotation

**Figure 2:**
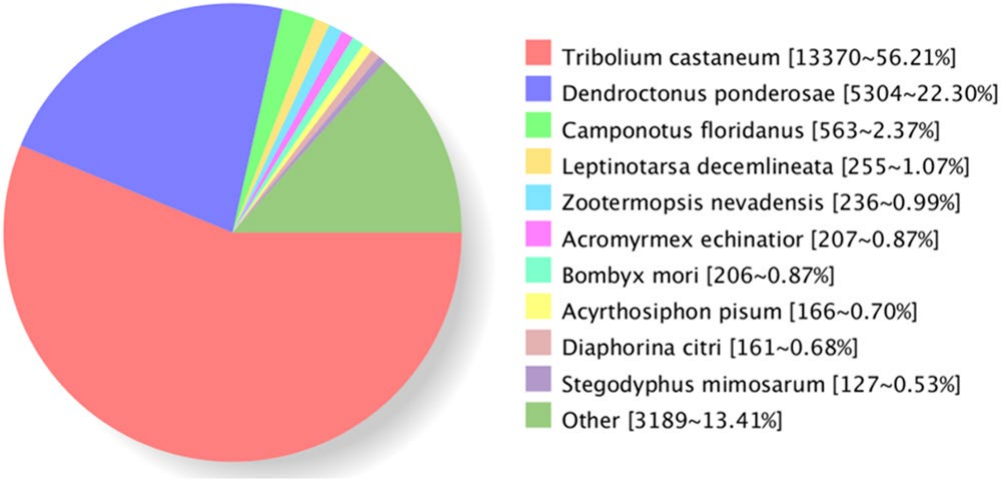
Homologous species distribution of *Agasicles hygrophila* annotated in the NR database.

NR is a non-redundant protein database in NCBI, which contains protein data from the Swiss-Prot, Protein Information Resource, Protein Research Foundation, Protein Data Bank, GenBank, and RefSeq.^21^ databases. The homologous species of *A. hygrophila* were predicted by sequence alignment on the basis of the NR database. Approximately 56.21% of sequences were aligned to *Tribolium castaneum*, followed by *Dendroctonus ponderosae* (22.3%) (Fig. 2).

### GO annotation

**Figure 3:**
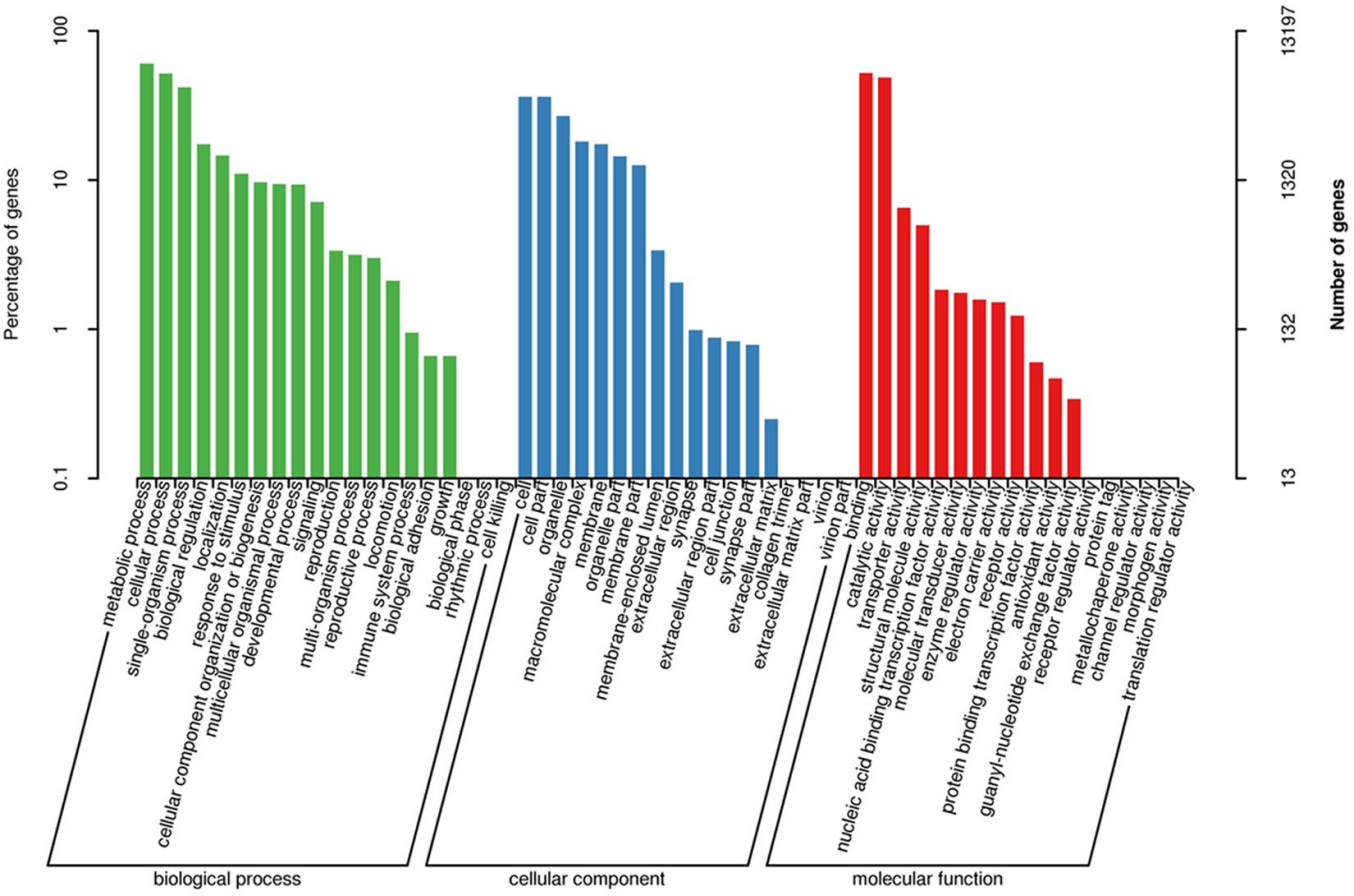
Gene Ontology (GO) functional annotation of *Agasicles hygrophila* transcripts. Green represents biological process; blue represents molecular function; and red represents cellular component. The x-axis represents GO categories; the y-axis (right) represents the number of transcripts; and the y-axis (left) represents the percentage of transcripts.

The GO database is produced by the Gene Ontology Consortium and features a structured, precisely defined, common, controlled vocabulary for describing the roles of genes and gene products in any organism. GO annotation system is a directed acyclic graph, including three categories: biological process (BP), molecular function (MF), and cellular component (CC). In this study, GO analysis revealed that the transcripts were enriched in several BP, MF, and CC associated terms (Fig. 3).

### COG annotation

**Figure 4:**
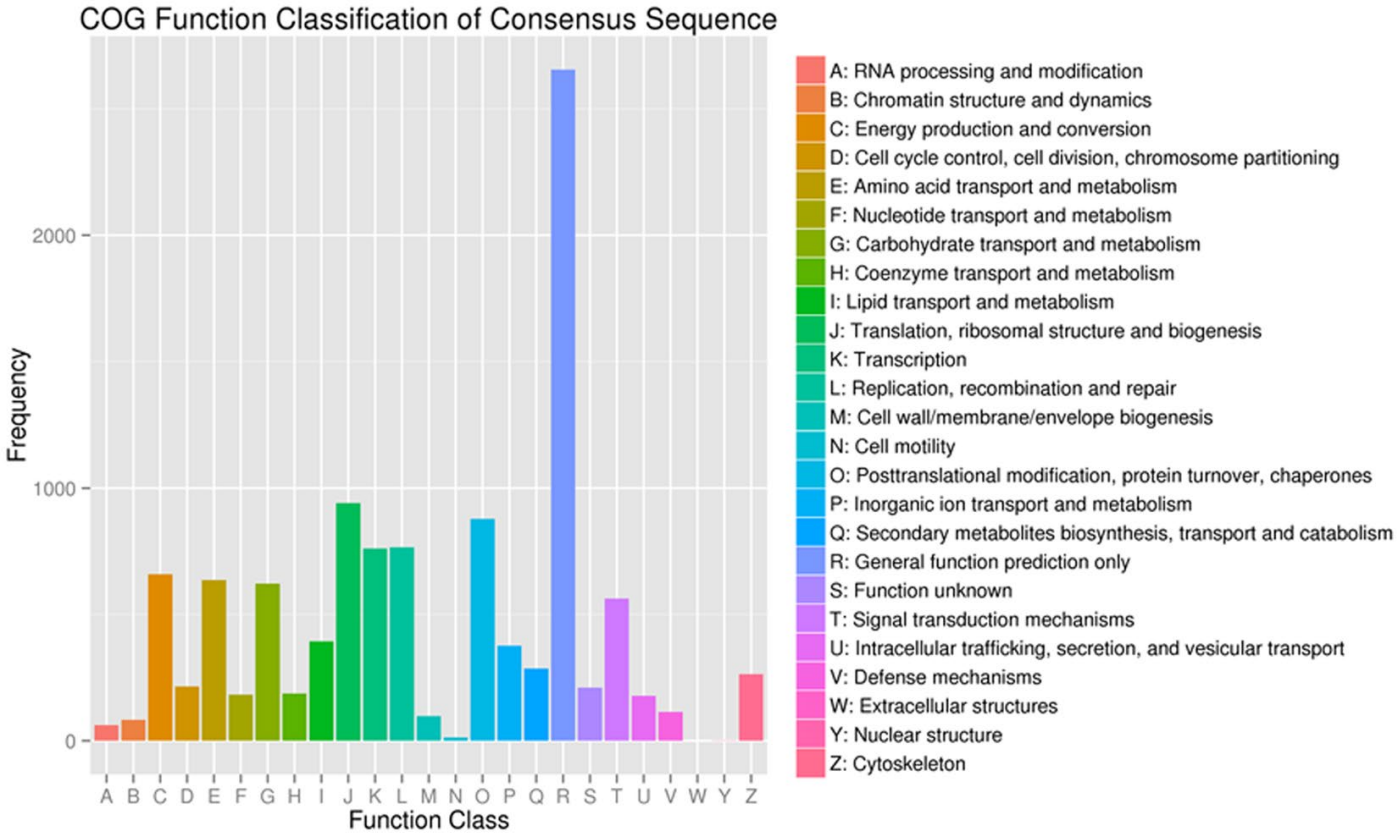
Clusters of Orthologous Groups of protein (COG) annotation of *Agasicles hygrophila* transcripts. The x-axis represents COG categories; the y-axis represents the number of transcripts.

The COG database is an attempt at phylogenetically classifying proteins encoded in 21 complete genomes of bacteria, archaea, and eukaryotes. The database can be used for the functional and phylogenetic annotation of newly sequenced genomes. This study also found that the number of transcripts that were enriched in function R was the most, followed by function J and function O (Fig. 4).

### lncRNA prediction

**Figure 5:**
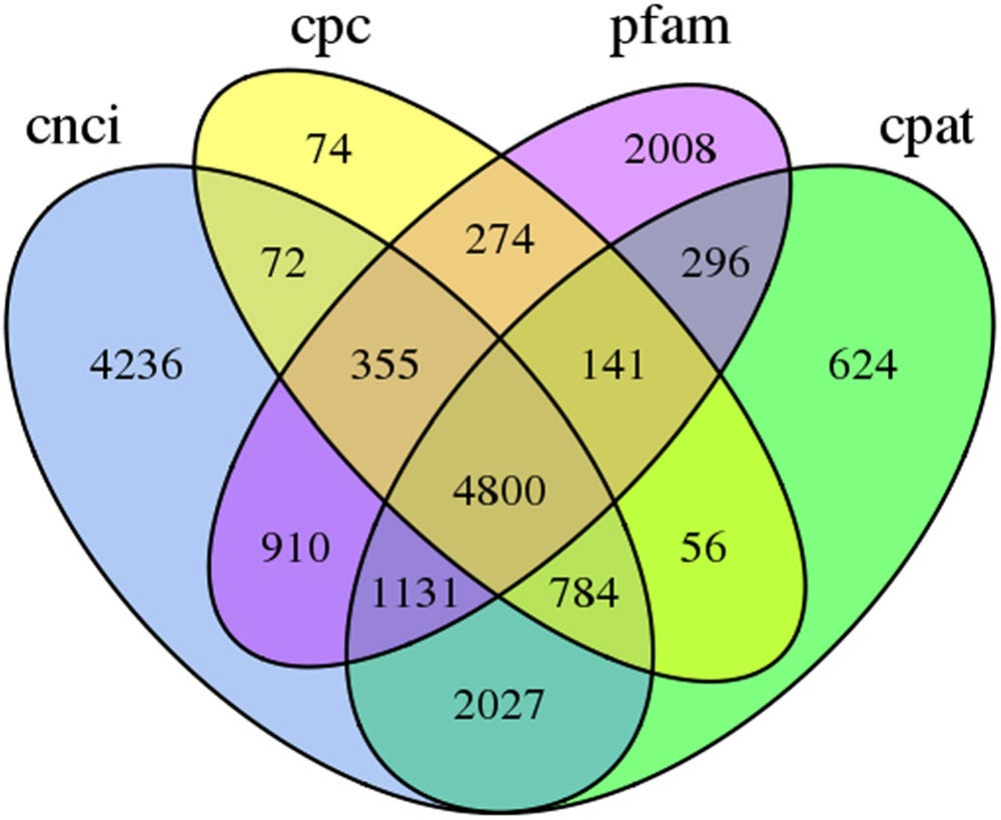
Venn diagram of the number of lncRNAs predicted by Calculator (CPC), Coding-Non-Coding Index (CNCI), Coding Potential Assessment Tool (CPAT), and pfam protein structure domain analysis.

The number of lncRNA transcripts, as predicted by CPC, CNCI, pfam protein structure domain analysis, and CPAT is shown in Fig. 5. In total, 4,198 lncRNA transcripts were predicted by all four methods.

## Discussion

The methodological strengths of SMRT sequencing have been comprehensively investigated in human^13^, which is superior to methods of short read sequencing due to the advantage of obtaining full-length transcripts. Besides, it could be used for the analysis of alternative splicing events, and the primary-percusor-mature RNAs structures to help better understand the RNA processing. In this study, 9.45 Gb of clean data were generated after SMRT sequencing, including 335,045 ROI and 158,085 FLNC reads. Total 40,004 consensus isoforms were identified through transcript clustering analysis of FLNC reads, which included 31,015 high-quality isoforms. After removing redundant sequences, 28,982 transcripts were obtained, and 145 alternative splicing events were predicted. SSR analysis revealed that 12,753 SSRs and 16,205 coding sequences were identified. Furthermore, 24,031 transcripts were annotated in eight functional databases. A total of 4,198 lncRNAs were predicted.

Based on 28,982 high-quality transcripts, a series of annotation analyses were performed. NR annotation revealed that 56.21% sequences were aligned to *T. castaneum*, followed by *D. ponderosae* (22.3%). *T. castaneum* is a member of the most species-rich eukaryotic order, an important model organism for studying generalized insect development^30^. Both *T. castaneum* and *D. ponderosae* belong to Coleoptera.

Genomic sequencing clearly revealed that the great majority of genes specifying the core biological functions are shared by all eukaryotes^31^. The rational classification of proteins encoded in sequenced genomes is critical for maximizing the use of genome sequences for functional and evolutionary studies^24^. In this study, these transcripts were enriched in various subcategories such as metabolic process, cellular process, cell, cell part, binding, and catalytic activity in the three main categories BP, MF, and CC according to the GO annotation analysis. The results of COG annotation showed that the largest number of transcripts were enriched in the function of general function prediction only. The results suggested that the transcripts of *A. hygrophila* were associated with the abovementioned functions.

LncRNAs, a novel class of nonprotein coding transcripts longer than 200 nt, are key regulatory molecules that can regulate gene expression at many different levels. Recently, increasing number of research has focused on the functions of lncRNAs in entomology, such as in *Drosophila melanogaster*, *Plutella xylostella*, and *Nilaparvata lugens*^32^, which provides a foundation for exploring the functions of lncRNA in insect development. This study identified 4,198 lncRNA transcripts with four analytical methods. However, their functions in *A. hygrophila* require further investigations.

In conclusion, our study, for the first time, completes SMRT sequencing of the full-length transcriptome of *A. hygrophila*. The obtained transcriptome may facilitate further studies on the genetic data of *A. hygrophila* and may help clarify the interactions between *A. hygrophila* and the ecosystem.

## Electronic supplementary material

Supplementary table 1
